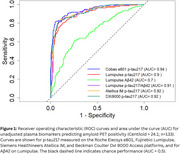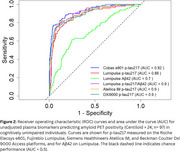# A comparison of four fully automated platforms for the measurement of plasma *p* ‐tau217 in the Alzheimer’s disease continuum

**DOI:** 10.1002/alz70861_108882

**Published:** 2025-12-23

**Authors:** Marisa N. Denkinger, Sterling C Johnson, Alpana Singh, James Liu, Kari Dieckhoff, Taina Marques, Rachael E. Wilson, Ramiro Eduardo Rea Reyes, Antoine Leuzy, Valentina Ghisays, Elizabeth Head, Thomas G Beach, Geidy E Serrano, Alireza Atri, Maria‐Magdalena Patru, Corey M. Carlson, Kinal Bhatt, Zivjena Vucetic, Arejas J. Uzgiris, Eddine Merabet, Robert C. Alexander, Reisa A. Sperling, Keith A. Johnson, Paul S. Aisen, Eric M. Reiman, Jessica B. Langbaum, Nicholas J. Ashton

**Affiliations:** ^1^ Banner Sun Health Research Institute, Sun City, AZ USA; ^2^ Wisconsin Alzheimer’s Disease Research Center, School of Medicine and Public Health, University of Wisconsin‐Madison, Madison, WI USA; ^3^ Wisconsin Alzheimer's Disease Research Center, University of Wisconsin‐Madison, School of Medicine and Public Health, Madison, WI USA; ^4^ Banner Alzheimer's Institute, Phoenix, AZ USA; ^5^ The UC Irvine Institute for Memory Impairments and Neurological Disorders, Irvine, CA USA; ^6^ Roche Diagnostics Corporation, Indianapolis, IN USA; ^7^ Beckman Coulter Inc., Chaska, MN USA; ^8^ Siemens Healthcare Laboratory, Berkeley, CA USA; ^9^ Center for Alzheimer Research and Treatment, Brigham and Women’s Hospital, Massachusetts General Hospital, Harvard Medical School, Boston, MA USA; ^10^ Center for Alzheimer Research and Treatment, Department of Neurology, Brigham and Women’s Hospital, Boston, MA USA; ^11^ Alzheimer's Therapeutic Research Institute, University of Southern California, San Diego, CA USA

## Abstract

**Background:**

Plasma *p* ‐tau217 outperforms other blood biomarkers for detecting Alzheimer’s disease (AD) pathology. To support clinical use and enable population‐level screening, assays must transition from research‐grade formats to fully automated, random‐access platforms that retain high diagnostic performance ‐ ensuring scalability, consistency, and broad applicability across healthcare and prevention settings. Currently, only one such fully automated assay has been reported^1^ but many are in development. This study compares four Research Use Only (RUO) assays on Lumipulse G1200, Roche cobas e801, Siemens Healthineers Atellica IM, and Beckman Coulter DxI 9000 Access platforms for plasma *p* ‐tau217 quantification in cognitively unimpaired (CU) and impaired (CI) individuals utilising amyloid PET as a standard of truth.

**Method:**

This study will include 5,574 plasma samples from 1,982 participants enrolled in the Wisconsin Registry for Alzheimer’s Prevention (WRAP). Of these, 1,746 individuals were cognitively unimpaired, 197 were cognitively impaired, and 676 participants underwent at least one or more amyloid PET scans. Plasma *p* ‐tau217 concentrations were measured using four fully automated immunoassay platforms: the Lumipulse G1200 and Roche cobas e801 assays were conducted at the Banner Sun Health Research Institute, while the Atellica IM and DxI 9000 platforms were evaluated at Siemens Healthineers and Beckman Coulter, respectively.

**Result:**

In this interim analysis, we evaluated 2,821 plasma samples across four RUO fully automated platforms. Using a 24.1 Centiloid (CL) amyloid PET cut‐off, cobas e801 showed an AUC of 0.94 (95% CI: 0.91–0.96; Figure 1), followed by Atellica IM and DxI 9000 (both AUC=0.92, 0.89–0.95). The Lumipulse *p* ‐tau217/Aβ42 ratio (AUC=0.91, 0.89–0.94) outperformed *p* ‐tau217 alone (AUC=0.90, 0.86–0.93). In cognitively unimpaired (CU) participants (Figure 2), cobas e801 had an AUC of 0.92 (0.89–0.96), while in cognitively impaired (CI) individuals, all assays showed similar performance (AUC >0.94).

**Conclusion:**

Fully automated immunoassays are essential for the widespread adoption of *p* ‐tau217, but they must uphold high diagnostic performances previously shown and recommended^2^. This study demonstrates four fully automated RUO assays exhibit comparable and desirable diagnostic accuracy for amyloid PET positivity. Further results will present findings from the full cohort (*n* =5,574), compare assays in terms of longitudinal change and relation to neuropathological change at post‐mortem.